# Effect of co-infection with intestinal parasites on COVID-19 severity: A prospective observational cohort study

**DOI:** 10.1016/j.eclinm.2021.101054

**Published:** 2021-07-31

**Authors:** Dawit Wolday, Teklay Gebrecherkos, Zekarias Gessesse Arefaine, Yazezew Kebede Kiros, Atsbeha Gebreegzabher, Geremew Tasew, Mahmud Abdulkader, Hiluf Ebuy Abraha, Abraham Aregay Desta, Ataklti Hailu, Getachew Tollera, Saro Abdella, Masresha Tesema, Ebba Abate, Kidist Lakew Endarge, Tsegaye Gebreyes Hundie, Frehiwot Kassahun Miteku, Britta C. Urban, Henk H.D.F. Schallig, Vanessa C. Harris, Tobias F. Rinke de Wit

**Affiliations:** a Mekelle University College of Health Sciences, Mekelle, Ethiopia; b Ethiopian Public Health institute, Addis Ababa, Ethiopia; c Tigray Health Research Institute, Mekelle, Ethiopia; d Eka Kotebe General Hospital, Addis Ababa, Ethiopia; e Department of Clinical Sciences, Respiratory Clinical Research Group, Liverpool School of Tropical Medicine, Liverpool, United Kingdom; f Department of Medical Microbiology and Infection Prevention, Experimental Parasitology Unit, Academic Medical Center, University of Amsterdam, Amsterdam, the Netherlands; g Amsterdam Institute of Global Health and Development, Department of Global Health, Amsterdam University Medical Center, Amsterdam, the Netherlands; h Joep Lange Institute, Amsterdam, the Netherlands

**Keywords:** COVID-19, Severity, parasite, africa, co-infection

## Abstract

*Background:* Severe acute respiratory syndrome coronavirus 2 (SARS-CoV-2) infection results in a spectrum of clinical presentations. Evidence from Africa indicates that significantly less COVID-19 patients suffer from serious symptoms than in the industrialized world. We and others previously postulated a partial explanation for this phenomenon, being a different, more activated immune system due to parasite infections. Here, we aimed to test this hypothesis by investigating a potential correlation of co-infection with parasites with COVID-19 severity in an endemic area in Africa.

*Methods:* Ethiopian COVID-19 patients were enrolled and screened for intestinal parasites, between July 2020 and March 2021. The primary outcome was the proportion of patients with severe COVID-19. Ordinal logistic regression models were used to estimate the association between parasite infection, and COVID-19 severity. Models were adjusted for sex, age, residence, education level, occupation, body mass index, and comorbidities.

*Findings:* 751 SARS-CoV-2 infected patients were enrolled, of whom 284 (37.8%) had intestinal parasitic infection. Only 27/255 (10.6%) severe COVID-19 patients were co-infected with intestinal parasites, while 257/496 (51.8%) non-severe COVID-19 patients were parasite positive (*p*<0.0001). Patients co-infected with parasites had lower odds of developing severe COVID-19, with an adjusted odds ratio (aOR) of 0.23 (95% CI 0.17–0.30; *p*<0.0001) for all parasites, aOR 0.37 ([95% CI 0.26–0.51]; *p*<0.0001) for protozoa, and aOR 0.26 ([95% CI 0.19–0.35]; *p*<0.0001) for helminths. When stratified by species, co-infection with *Entamoeba* spp., *Hymenolopis nana, Schistosoma mansoni*, and *Trichuris trichiura* implied lower probability of developing severe COVID-19. There were 11 deaths (1.5%), and all were among patients without parasites (*p* = 0.009).

*Interpretation:* Parasite co-infection is associated with a reduced risk of severe COVID-19 in African patients. Parasite-driven immunomodulatory responses may mute hyper-inflammation associated with severe COVID-19.

*Funding:* European and Developing Countries Clinical Trials Partnership (EDCTP) – European Union, and Joep Lange Institute (JLI), The Netherlands.

*Trial registration:* Clinicaltrials.gov: NCT04473365

**Research in context**

**Evidence before this study**

We searched PubMed, medRxvi, and bioRxiv for [“parasite” OR “helminth” OR “Protozoa”] AND [“COVID-19″ OR “SARS-CoV-2″] until April 12, 2021, with no language restriction. Ecological studies reported an inverse correlation between incidence of COVID-19 and parasites, like with soil-transmitted helminths, schistosomiasis, or malaria. Some reports proposed that helminth co-infection may modulate, or mute COVID-19 severity in endemic regions. To the best of our knowledge, there are no reports published to date actually demonstrating a clear association between parasite co-infection and COVID-19 severity.

**Added value of this study**

In this unique cohort study consisting of 751 Ethiopian patients, we determined the association between co-infection with parasites and COVID-19 severity. We identified 284 (37.8%) COVID-19 patients to be co-infected with one or more parasites. We demonstrate that the proportion of COVID-19 patients with parasitic infection decreased with increasing categories of disease severity. When stratified by species-specific parasite co-infection status, we observed same trends. Using a multivariable logistic regression analysis, and after adjusting for sex, age, residence, and the presence of comorbidities, we observed that co-infection with any parasite, including protozoa, or helminths, was associated with lower odds of developing severe COVID-19. In addition, patients without parasite co-infection appeared vulnerable to worse in-hospital outcomes, including admission to the intensive-care unit, requirement for supplemental oxygen, or mechanical ventilation, and death.

**Implications of all the available evidence**

Co-infection with parasites is associated with reduced development of severe COVID-19 in this Africa setting. Our findings confirm the hypothesis that co-infection with parasites may mute hyper-inflammation associated with severe COVID-19. Further research is needed to unravel underlying immunology and consequences for SARS-CoV-2 vaccination in Africa. Implications might involve COVID-19 vaccine efficacy in Africa.

Alt-text: Unlabelled box

## 1 Introduction

Infection with severe acute respiratory syndrome coronavirus 2 (SARS-CoV-2) results in a spectrum of clinical presentations. Whereas most people with COVID-19 develop asymptomatic or mild illness, at-risk patients can develop severe pneumonia and hypoxemia disease requiring hospitalization [1], [2], [3]. In severe cases, COVID-19 can be complicated by acute respiratory distress syndrome (ARDS), sepsis, multi-organ failure, including acute kidney injury, and cardiac injury [2], [3], [4], [5]. Risk factors for a severe disease course and mortality due to COVID-19 include older age, immune compromise, and underlying co-morbidities, particularly non-communicable diseases (NCDs), such as hypertension, cardiovascular disease and diabetes [2], [3], [4], [5].

Low and medium-income countries (LMICs) differ significantly in disease prevalence and conditions from high-income countries (HICs). Infectious diseases have a markedly higher prevalence in LMICs, including so-called neglected infectious diseases (NIDs). Amongst NIDs, parasitic infections affect more than 2 billion people throughout the world, with disproportionately high prevalence rates in resource-poor settings [6,7]. Multicellular and highly complex parasites such as *Ascaris*, hook worm, *Trichuris, Enterobius,* and *Schistosoma*, as well as unicellular organisms including *Entamoeba, Giardia, Toxoplasma*, Cyclospora and Cryptosporidia are among the major organisms that contribute to the global intestinal parasitic disease burden [6,7].

Chronic and/or persistent parasitic infections are common in LIMCs, and such chronic infections, possibly in part through direct modulation of the host's immune responses, were shown to alter clinical outcomes to other infections [8,9]. Pre-existing parasitic infections may also modify the host's immune response to infection with SARS-CoV-2, with postulated beneficial and detrimental effects [10], [11], [12], [13]. To the best of our knowledge, no studies to date have assessed the possible association between parasitic co-infection and COVID-19 disease severity. We and other previously hypothesized parasite infections to skew the immune system towards TH2 responses, thus precluding TH1 hyper immune activation that is characteristic of COVID-19 severity [11], [12], [13]. The objective of this study was to test this hypothesis by comparing the parasitic infections of COVID-19 patients stratified for clinical outcomes.

## 2 Methods

### 2.1 Study design and participants

This study is part of Profile-CoV project (Clinicaltrials.gov: NCT04473365), a prospective observational cohort study being undertaken in two sites in Ethiopia, with the aim of profiling of immunological response to SARS-CoV-2 in the context of persistent immune activation in Sub-Saharan Africa. This study included individuals who were recruited for Profile-CoV and subsequently screened them for intestinal parasitic infections.

### 2.2 Procedures

Individuals presenting to the Kuyha (Mekelle University College of Health Sciences, Mekelle), and Eka Generalized Hospital (Addis Ababa) who qualified for testing were screened for SARS-CoV-2 infection with a nasopharyngeal swab, and real-time polymerase chain reaction (RT-PCR). Following the declaration by the WHO that COVID-19 became pandemic, the Ethiopian Ministry of Health implemented a mass screening of all travelers, people who had come in contact with COVID-19 patients, those from high risk settings (e.g. health-care workers), as well as those with symptoms suggestive of COVID-19. All patients with confirmed SARS-CoV-2 infection were admitted to dedicated COVID-19 Isolation and Treatment Centers. Patients were admitted irrespective of clinical severity status. Whereas 515 patients were included from Kuyha Hospital in Mekelle between July and October 2020, the 236 cases from Eka Generalized Hospital in Addis Ababa were enrolled between February and March 2021. Admitted patients, if symptomatic, received supportive therapy according to their clinical need. Patients with severe disease received high-flow oxygen via nasal cannula or intubation as well as dexamethasone. Whereas patients admitted to the intensive care unit (ICU) were followed every day, or as needed, those with asymptomatic or mild/moderate clinical presentation were quarantined, and followed every 3–5 days, or as needed up until discharge.

Sociodemographic, clinical, and laboratory data were collected using standardized Case Record Forms (CRFs) adapted from the International Severe Acute Respiratory and Emerging Infection Consortium's (ISARIC) CRFs for emerging severe acute respiratory infections [14]. Patient's clinical status was stratified following the WHO criteria as asymptomatic, mild/moderate, severe (with dyspnea, respiratory rate ≥ 30 breaths per minute, O2 saturation ≤ 93%, lung infiltrates ≥ 50% of the lung fields within 24–48 h), and critical (with respiratory failure, septic shock, and/or multiple organ failure) [15]. All data were then entered into electronic medical records.

SARS-CoV-2 infection was confirmed by RT-PCR on samples obtained from nasopharyngeal swabs, according to manufacturer's instructions (TIB Molbiol, Berlin, Germany). Fresh stool sample specimens were obtained for examination for parasites and ova. Analysis included direct microscopic examination and modified Ritchie concentration method [16]. In addition, the intensity of infection was determined using Kato-Katz method and was calculated and reported as individuals’ eggs per gram of feces (EPG), as described previously [16], and recommended by the WHO [17]. All patients who were positive for intestinal parasites received parasite-specific therapy. None of these patients received ivermectin.

The study protocol was reviewed and approved by the Health Research Ethics Review Committee of Mekelle University College of Health Sciences (No.: ERC 1769/2020), the Ethiopian Public Health Institute (No.: EPHI 6.13/814), and Eka Kotebe General Hospital (No.: EK/150/5/32). Written informed consent was obtained by all participants, or their guardians, for participation in the study.

### 2.3 Outcomes

The primary outcome for this study was the proportion of severe COVID-19 among SARS-CoV-2 positive patients with and without a parasitic co-infection. Asymptomatic and mild/moderate COVID-19 cases were classified as non-severe cases and severe and critical COVID-19 cases were classified as severe. Secondary outcomes included requirement for supplemental oxygen, and/or mechanical ventilation, admission to ICU, and death.

### 2.4 Statistical analysis

We hypothesized that co-infection with intestinal parasite is associated with reduced risk of severe COVID-19 [13]. Exposure was intestinal parasite co-infection. The primary outcome of the study was the proportion of severe disease among SARS-CoV-2 PCR positive patients. A feasibility study showed that about 20% of COVID-19 patients with parasite co-infection developed severe COVID-19. We expected this to increase to about 50% among those without parasite co-infection. A minimum sample size of 223 patients with parasite, and 446 without parasite co-infection (at 1:2 ratio) was required to ensure 80% power using a two-sided test with a significance level of α=0.05. Assuming a dropout rate of 10%, a minimum total sample size of 738 was estimated for the cohort.

Baseline characteristics for continuous variables were expressed as the median with interquartile range (IQR), and for categorical variables as proportions. Whereas categorical variables were compared using χ² test or Fisher's exact test, continuous variables were compared by Mann-Whitney U, or Kruskal-Wallis tests, as appropriate. Normality of distribution of variables was ascertained by running Wilk test of normality, before analysis by Mann-Whitney U, or Kruskal-Wallis tests. The association between COVID-19 severity and parasitic co-infection was determined by ordinal logistic regression analysis. Independent variables, including age, sex, residence, comorbidity, parasite infection (overall and dis-aggregated by parasite type into protozoa, helminths, and species level), were included in the initial univariate analysis. We included sex, age, education level, occupation, pregnancy, body mass index and comorbidities as confounding factors as they have been associated with poor clinical outcomes among patients with COVID-19 [2], [3], [4], [5]. In addition, we adjusted for urban vs. rural residence as this has been shown to influence differential immune responses [18]. A multivariate regression analyses [adjusted odds ratio (aOR)] were calculated by including all variables that were *p*<0.05 by univariate analysis. *P* values <0.05 were considered statistically significant. Data was analyzed using STATA (Statistical package v. 14.0, StataCorp, Texas, USA). Reporting of this study was undertaken according to the STROBE statement (Supplemental Table 4).

### 2.5 Role of funding source

Funders had no role in the study design, study participant selection, and recruitment, data collection, analysis, data interpretation, decision to publish, or preparation of the manuscript.

## 3 Results

### 3.1 Study population characteristics

**Fig. 1:**
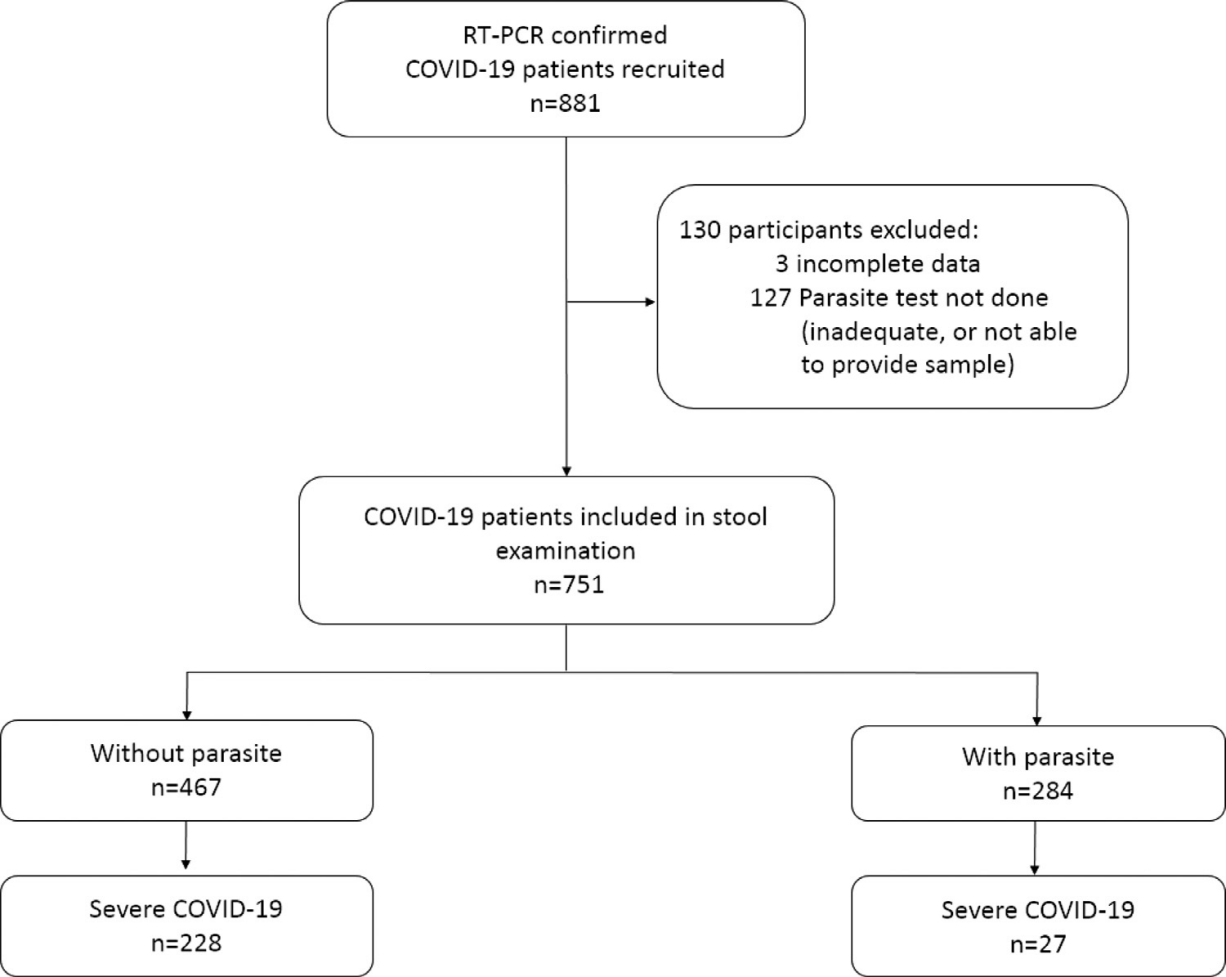
Flow diagram of study participants.

A total of 881 COVID-19 cases were recruited and 130 were excluded, either due to incomplete data or inadequate stool samples, or patient not able to provide sample (Fig. 1). Baseline socio-demographic data of the study participants is summarized in Table 1. The majority of the study population was male (63.9%). The median age of the cohort was 37 (IQR 28–50; range 3–92) years, with 53.0% being in the age range of 24 to 44 years. Those older than 60 years and above comprised only 16.1%. Patients were hospitalized for a median of 12 days (range: 3 to 45 days). Notably, 36.2% of the cohort was asymptomatic, and 29.8% had mild/moderate symptoms at the time of diagnosis; the remaining 29.0% and 4.9% had severe, and critical disease (requiring admission to the ICU), respectively (Supplemental Table 1).

**Table 1:** Clinical features among COVID-19 patients without, or with parasite co-infection.

Characteristic	All patients *n* = 751	Without parasite *n* = 467	With parasite *n* = 284	p-Value
**Socio–demographic features:**				
Gender				
Male	480 (63.9%)	307 (65.7%)	173 (60.9%)	0.011
Female (not pregnant)	257 (34.2%)	147 (31.5%)	110 (38.7%)	
Female (pregnant)	14 (1.9%)	13 (2.8%)	1 (0.4%)	
Age in years	37 (28–50)	40 (30–57)	32 (25–42)	0.064
Age group [years]				
< 24	92 (12.3%)	42 (9.0%)	50 (17.6%)	0.0001
24 – 44	398 (53.0%)	224 (48.0%)	174 (61.3%)	
45 – 59	140 (18.6%)	99 (21.2%)	41 (14.4%)	
≥ 60	121 (16.1%)	102 (21.8%)	19 (6.7%)	
Residence				
Rural	141(18.8%)	68 (14.6%)	73 (25.7%)	0.0001
Urban	593 (79.0%)	393 (84.2%)	200 (70.4%)	
Undetermined	17 (2.3%)	6 (1.3%)	11 (3.9%)	
Education level				
≤ Primary	215 (28.6%)	124 (26.5%)	91 (32.0%)	0.210
Secondary	203 (27.0%)	126 (27.0%)	77 (27.1%)	
College/university	333 (44.3%)	217 (46.5%)	116 (40.9%)	
Occupation				
Unemployed	250 (33.3%)	149 (31.9%)	101 (35.6%)	0.587
Employed	421 (56.0%)	267 (57.2%)	154 (54.2%)	
Health care workers	80 (10.7%)	51 (10.9%)	29 (10.2%)	
**Clinical symptoms and signs:**				
Fever	248 (33.0%)	200 (42.8%)	48 (16.9%)	<0.0001
Dyspnea	199 (26.5%)	176 (37.7%)	23 (8.1%)	<0.0001
Cough (any type)	339 (45.1%)	259 (55.5%)	80 (28.2%)	<0.0001
Non–productive cough	148 (19.7%)	94 (20.1%)	54 (19.0%)	0.0001
Productive cough	191 (25.4%)	165 (35.3%)	26 (9.2%)	
Hemoptysis	21 (2.8%)	19 (4.1%)	2 (0.7%)	0.007
Chest pain	47 (6.3%)	39 (8.4%)	8 (2.8%)	0.002
Sore throat	70 (9.3%)	54 (11.6%)	16 (5.6%)	0.007
Head ache	223 (29.7%)	181 (38.8%)	42 (14.8%)	<0.0001
Nasal congestion	42 (5.6%)	23 (4.9%)	19 (6.7%)	0.307
Loss of smell and/or taste	59 (7.9%)	36 (7.7%)	23 (8.1%)	0.847
Nausea/vomiting	76 (10.1%)	67 (14.4%)	9 (3.2%)	<0.0001
Abdominal pain	44 (5.9%)	35 (7.5%)	9 (3.2%)	0.014
Diarrhea	39 (5.2%)	32 (6.9%)	7 (2.5%)	0.009
Myalgia	112 (14.9%)	98 (21.0%)	14 (4.9%)	<0.0001
Body mass index				
18.5–24.9	668 (88.9%)	399 (85.4%)	269 (94.7%)	<0.0001
<18.5	4 (0.5%)	3 (0.6%)	1 (0.4%)	
25.0–29.9	56 (7.5%)	42 (9.0)	14 (4.9%)	
≥30.0	23 (3.1%)	23 (4.9%)	0 (0.0%)	
Temperature > 37.3 °C	50 (6.7%)	41 (8.8%)	9 (3.2%)	0.003
Temperature	36.0 (36.0–36.9)	36.2 (36.0–37.0)	36.0 (36–36.7)	<0.00001
Systolic blood pressure (mmHg)	120 (110–130)	120 (110–130)	115 (110–125)	0.0009
Diastolic blood pressure (mmHg)	75 (68–80)	75 (68–80)	75 (68–80)	0.8395
Respiratory rate (breaths/minute)	22 (20–25)	23 (21–28)	22 (19–23)	<0.00001
Heart rate (beats/minute)	85 (76–94)	86 (78–96)	83 (76–90)	<0.00001
Laboratory data*				
Lymphocyte, x10^9^/L	1.2 (0.8–1.6)	1.2 (0.8–1.6)	1.3 (0.9–1.7)	0.7008
Hematocrit,%	44.0 (40.1–46.9)	44.2 (40.6–47.4)	42.4 (38.2–45.7)	0.0296
Platelet count, x10^9^ /L	217 (163–294)	215 (159–288)	247(204–334)	0.0488
Alanine aminotransferase concentration, U/L	40 (25–67)	40 (25–67)	48 (36–55)	0.6841
Creatinine concentration, mg/dL	0.87 (0.68–1.04)	0.88 (0.70–1.04)	0.62 (0.48–1.47)	0.0135
**Comorbidities**				
Comorbidity (at least 1)	216 (28.8%)	178 (38.1%)	38 (13.4%)	<0.0001
Non–communicable disease (NCDs) comorbidities	179 (23.8%)	150 (32.1%)	29 (10.2%)	<0.0001
Diabetes	97 (12.9%)	84 (18.0%)	13 (4.6%)	<0.0001
Hypertension	87 (11.6%)	74 (15.9%)	4.6 (4.5%)	<0.0001
Cardio vascular diseases	19 (2.5%)	18 (3.9%)	1 (0.4%)	0.003
Chronic obstructive lung diseases and asthma	23 (3.1%)	19 (4.1%)	4 (1.4%)	0.040
Chronic liver disease	8 (1.1%)	8 (1.7%)	0 (0.0%)	0.027
Chronic kidney disease	11 (1.5%)	9 (1.9%)	2 (0.7%)	0.176
Surgery cases	15 (2.0%)	12 (2.6%)	3 (1.1%)	0.151
Communicable disease comorbidities				
HIV	17 (2.3%)	14 (0.3.0%)	3 (1.1%)	0.083
Tuberculosis	1 (0.1%)	1 (0.2%)	0 (0.0%)	0.435
**Outcomes**				
Admission to ICU	56 (7.5%)	44 (9.4%)	12 (4.2%)	0.009
Supplemental oxygen	243 (32.4%)	216 (46.3%)	27 (9.5%)	<0.0001
Invasive mechanical ventilation	53 (7.1%)	46 (9.9%)	7 (2.5%)	<0.0001
Death	11 (1.5%)	11 (2.4%)	0 (0.0%)	0.009
Severe COVID–19	255 (34.0%)	228 (48.8%)	27 (9.5%)	<0.0001

Data are expressed as n (%) or median (IQR). p values are from χ² test, or Fisher's Exact test (for categorical variables), and Mann-Whitney U, or Kruskal-Wallis tests (for continuous variables), as appropriate.

*Data missing for 490, 487, 492, 622, and 540 patients for lymphocyte, hematocrit, platelet, alanine aminotransferase, and creatinine concentrations, respectively.

Patients with severe, or critical disease at presentation were older and had more symptoms, including fever, dyspnoea, cough, hemoptysis, chest pain, sore throat, head ache, nausea/vomiting, abdominal pain, diarrhea, and myalgia, when compared to those presenting with asymptomatic, or mild/moderate clinical status (Supplemental Table 1). Nasal congestion, and loss of smell/taste was reported less frequently in severe, or critical cases. Co-morbid conditions were significantly higher among severe, and critical COVID-19 cases when compared to non-severe cases (Supplemental Table 1). In addition, COVID-19 patients with NCDs were older, the majority (45.3%) being ≥ 60 years old (*p* = 0.0001), and more symptomatic (Supplemental Table 2). Severe/critical clinical manifestation was more frequent in COVID-19 patients with NCDs compared to those without NCDs [(*p*<0.0001), Supplemental Table 2].

### 3.2 Prevalence of intestinal parasites

Of the total 751 individuals enrolled in the study, 284 (37.8%) harbored one or more intestinal parasites (Table 2). Of the patients included in the study, protozoa and helminth infections comprised 20.2% and 24.5%, respectively. The most common protozoa infections were *Entamoeba* spp. (16.8%) and *Giardia* (3.6%). For helminths, the most common infections were *H. nana* (15.1%), *S. mansoni* (4.5%), and *A. lumbricoides* (3.6%). The proportion of patients harboring multiple parasites (>1) was 8.7% (Table 2). There was no significant difference in sex and age distribution between those with parasites and those without (Table 1). However, those without parasite co-infection appeared more symptomatic for COVID-19. In addition, the proportion of comorbid conditions, in particular NCDs, was significantly higher in COVID-19 patients without parasitic co-infection (Table 1).

**Table 2:** Prevalence of parasitic infections in the different COVID–19 severity category.

Parasite co–infection	All patients (*n* = 751)	COVID-19 severity category				p-Value
		Asymptomatic (*n* = 272)	Mild/moderate (*n* = 224)	Severe (*n* = 218)	Critical (*n* = 37)	
Any parasite	284 (37.8%)	150 (55.2%)	107 (47.8%)	22 (10.1%)	5 (13.5%)	<0.0001
Protozoa – all	152 (20.2%)	77 (28.3%)	59 (26.3%)	12 (56.5%)	4 (10.8%)	<0.0001
*Entamoeba* spp. *cyst*	109 (14.5%)	63 (23.2%)	38 (17.0%)	6 (2.8%)	2 (5.4%)	<0.0001
*Entamoeba histolytica trophozoite*	17 (2.3%)	5 (1.8%)	10 (4.5%)	2 (0.9%)	0 (0.0%)	0.087
*Giardia lamblia cyst*	12 (1.6%)	4 (1.5%)	4 (1.5%)	2 (0.9%)	2 (5.4%)	0.227
*Giardia lamblia trophozoite*	15 (2.0%)	6 (2.2%)	7 (3.1%)	2 (0.9%)	0 (0.0%)	0.393
Helminth – all	184 (24.5%)	103 (37.9%)	68 (30.4%)	12 (5.5%)	1 (2.7%)	<0.0001
*Hymenolopis nana*	113 (15.1%)	59 (21.7%)	47 (21.0%)	7 (3.2%)	0 (0.0%)	<0.0001
*Schistosoma mansoni*	34 (4.5%)	22 (8.1%)	10 (4.5%)	2 (0.9%)	0 (0.0)	0.001
*Ascaris lumbricoides*	27 (3.6%)	13 (4.8%)	10 (4.5)	3 (1.4%)	1 (2.7%)	0.142
*Trichuris trichiura*	11 (1.5%)	8 (2.9%)	3 (1.3%)	0 (0.0%)	0 (0.0%)	0.050
Hook worm	12 (1.6%)	8 (2.9%)	3 (1.3%)	1 (0.5%)	0 (0.0%)	0.169
*Taenia* spp.	3 (0.4%)	3 (1.1%)	0 (0.0%)	0 (0.0%)	0 (0.0%)	0.239
Soil–transmitted helminths only	48 (6.4%)	26 (9.6%)	17 (7.6%)	4 (1.8%)	1 (2.7%)	0.002
Poly–parasitism – any	65 (8.7%)	37 (13.6%)	25 (11.2%)	3 (1.4%)	0 (0.0%)	<0.0001
Protozoa plus helminth	50 (6.7%)	30 (11.0%)	18 (8.1%)	2 (0.9%)	0 (0.0%)	<0.0001
Helminth plus helminth	19 (2.5%)	11 (4.0%)	7 (3.1%)	1 (0.5%)	0 (0.0%)	0.046

Data are expressed as n (%). P values are from χ² test, or Fisher's Exact test, as appropriate.

### 3.3 Association of parasitic co-infection with COVID-19 severity

The proportion of severe, or critical COVID-19 was significantly higher in patients without parasitic co-infection (Table 1). The proportion of severe COVID-19 in patients without parasites (196/467 [42.0%, CI 37.56–46.52]) was significantly higher than in those with parasites (22/284 [7.8%, CI 5.14–11.51]); *p*<0.0001. Likewise, the proportion of critical COVID-19 in patients without any parasite infection (32/467 [6.9%, CI: 4.88–9.54]) was significantly higher than in those co-infected with parasites (5/284 [1.8, CI 0.73–4.18]; *p*<0.0001). The proportion of severe COVID-19 was higher in the in the non-protozoa group (206/599 [34.4%, CI 30.68–38.30]) when compared to the protozoa group (12/152 [7.9%, CI 4.51–13.47]; *p*<0.0001), and in the helminth negative group (206/567 [36.3%, CI 32.46–40.39]) compared to the helminth co-infected group (12/184 [6.5%, CI 3.72–11.19]; *p* = 0.0001). Furthermore, the proportion of critical COVID-19 cases was higher in the in the non-protozoa group (33/599 [5.5%, CI 3.94–7.66]) when compared to the protozoa group (4/152 [2.6%, CI 0.98–6.87]; *p*<0.0001), and in the helminth negative group (36/567 [6.4%, CI 4.61–8.69]) compared to the helminth co-infected group (1/184 [0.5%, CI 0.08–3.82]; *p* = 0.0001). We did not observe any correlation between helminth egg-load and COVID-19 severity.

**Fig. 2:**
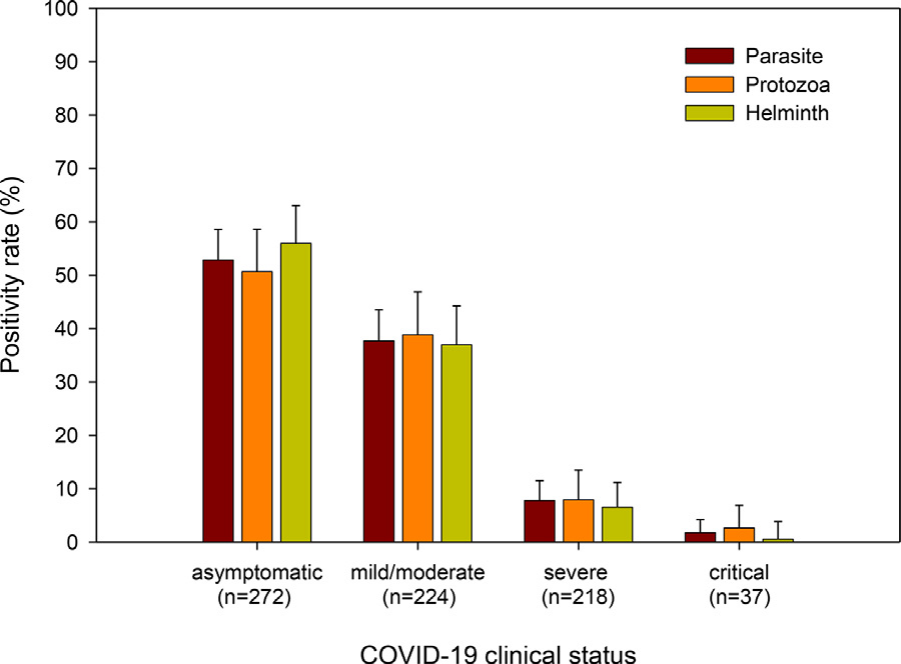
Relationship of parasite co-infection with categories of COVID-19 severity. Proportion of patients with different categories of COVID-19 severity co-infected with any parasite, protozoa, or helminths. Error bars indicate 95% CI. *p*<0.0001 (for parasite, and protozoa), and *p* = 0.001 (for helminths) by Kruskal-Wallis rank test.

Thus, parasitic infections were inversely correlated with COVID-19 severity. A higher proportion of patients without parasitic co-infection were admitted to the ICU, required supplemental oxygen, and/or mechanical ventilation, or died compared to those with parasitic co-infection (Table 1). The proportion of COVID-19 patients with any parasite infection, or protozoa, or helminth infection decreased with increasing categories of disease severity (Table 2; Fig. 2). When stratified by species-specific parasite co-infection status, we observed similar trends with significant decrease in COVID-19 severity for those co-infected with poly-parasites, soil-transmitted helminths, *Entamoeba* spp. cyst, *H. nana, S. mansoni*, and *T. trichiura* compared to those without (Supplemental Fig. 1).

**Fig. 3:**
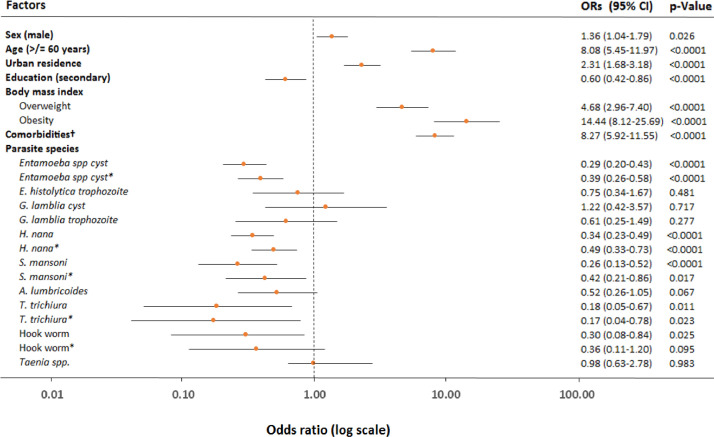
ORs for the association between species-specific parasite co-infection and COVID-19 severity. All models and those stratified by species-specific parasitic co-infection were analysed by univariable ordinal logistic regression analysis. *Multivariable models adjusted for sex, age, residence, education, body mass index, and comorbidities† (including diabetes, hypertension, cardio-vascular diseases, and chronic liver diseases). ORs=odds ratios. Lines are 95% CIs.

Ordinal logistic regression analysis was used in order to ascertain the association between parasite infection and COVID-19 severity. In univariate analysis, male sex, age ≥ 60 years, urban residence, primary education level, being overweight, or obese, and comorbidities, in particular, NCDs including diabetes, hypertension, cardio-vascular diseases, and chronic liver disease were all associated with increased odds of severe COVID-19 (Table 3). However, occupation, pregnancy, chronic obstructive lung disease, including asthma, chronic kidney disease, surgery, and HIV-1 infection appeared not associated with severe COVID-19. In contrast, having any parasitic, protozoal, or helminth infection was associated with lower odds of developing severe COVID-19. In multivariate analysis, age ≥ 60 years, urban residence, obesity, comorbidity, in particular NCDs including hypertension, and chronic liver disease were associated with increased odds of severe COVID-19 (Table 3). After adjustment for sex, age, residence, obesity, and the presence of comorbidities, COVID-19 patients with any parasitic co-infection (aOR 0.35 [95% CI 0.26–0.48]; *p*<0.0001), protozoal co-infection (aOR 0.51 [95% CI 0.36–0.73]; *p*<0.0001), as well as helminth co-infection (aOR 0.37 [95% CI 0.27–0.52]; *p*<0.0001) had a lower odds of developing severe COVID-19 when compared to patients without a parasitic co-infection (Table 3). We also noted that harboring multiple parasites was strongly associated with less severe COVID-19 (aOR 0.43 [95% CI 0.26–0.71]; *p*<0.0001; Table 3). Further analysis stratified by parasite species level revealed that patients co-infected with *Entamoeba cyst spp* (aOR 0.39 [95% CI 0.26–0.58]; *p*<0.0001), or *Hymenolopis nana* (aOR 0.49 [95% CI 0.33–0.73]; *p*<0.0001), or *Schistosoma mansoni* (aOR 0.42 [95% CI 0.21–0.86; *p* = 0.017)], or *T. trichiura* (aOR 0.17 [95% CI 0.04–0.78); *p* = 0.023) co-infection tended to have lower odds of developing severe COVID-19 (Fig. 3). Covid-19 patients co-infected with Hook worm also tended to exhibit decreased risk of severe disease, but this was not statistically significant (Fig. 3). Smaller sample sizes precluded observations for the remaining parasite species. We could not determine association between death (*n* = 11), and (lack-of) co-infection with parasites because of the small number of death outcomes. Notably, after adjusting for sex, age, residence, pregnancy, education, occupation, and body mass index we found that the odds of having a NCD was significantly lower in COVID-19 patients with parasitic co-infection (aOR 0.35 [95% CI 0.21–0.60], *p*<0.0001), or helminth infection (aOR 0.21 [95% CI 0.10–0.43]; *p*<0.0001; Supplemental Table 3).

**Table 3:** Factors associated with severe COVID–19.

Characteristic	Univariable model		Multivariable model	
	Unadjusted OR (95% CI)	p-Value	Adjusted OR (95% CI)	p-Value
Gender				
Male	1	..	1	..
Female (not pregnant)	0.70 (0.53–0.92)	0.011	0.86 (0.64–1.15)	0.313
Female (pregnant)	1.72 (0.69–4.28)	0.242	2.09 (0.79–5.59)	0.139
				
Age (≥60 years vs. <60)	8.08 (5.45–11.97)	<0.0001	3.41 (2.13–5.46)	<0.0001
Rural vs. urban residence	0.38 (0.28–0.516)	<0.0001	0.52 (0.38–0.72)	<0.0001
Education level				
≤ Primary	1	..	1	..
Secondary	0.60 (0.42–0.86)	0.006	1.02 (0.68–1.54)	0.911
College/university	0.91 (0.66–1.25)	0.569	..	..
Occupation				
Unemployed	1	..	1	..
Employed	1.01 (0.76–1.35)	0.939	..	..
Health care workers	1.26 (0.81–1.94)	0.304	..	..
Body mass index				
18.5–24.9	1	..	1	..
<18.5	0.52 (0.04–6.34)	0.610	..	..
25.0–29.9	4.68 (2.96 –7.40)	<0.0001	1.34 (0.61–2.94)	0.460
≥30.0	14.44 (8.12–25.69)	<0.0001	2.68 (1.22–5.87)	0.014
Comorbidity (at least 1)*	8.27 (5.92–11.55)	<0.0001	5.43 (3.50–48.42)	<0.0001
Non–communicable disease (NCDs) comorbidities*	10.58 (7.36–15.20)	<0.0001	7.74 (4.64–12.90)	<0.0001
Diabetes	6.41 (4.22–9.76)	<0.0001	1.61 (0.78–3.32)	0.198
Hypertension	9.63 (6.10–15.18)	<0.0001	2.87 (1.63–5.907)	<0.0001
Cardio vascular diseases	5.27 (2.37–11.74)	<0.0001	1.25 (0.504–3.12)	0.633
Chronic obstructive lung diseases	1.90 (0.90–4.04)	0.093	..	..
Chronic kidney disease	2.13 (0.72–6.74)	0.174	..	..
Chronic liver disease	3.57 (1.0.8–11.78)	0.037	4.67 (1.30–16.82)	0.018
Surgery cases	0.64 (0.25–1.66)	0.362	..	..
Communicable disease comorbidities				
HIV	2.05 (0.83–5.07)	0.121		..
Tuberculosis	1.82e+09 (..–..)	0.998	..	..
Parasite co–infection**				
Any parasite (at least 1)	0.23 (0.17–0.30)	<0.0001	0.35 (0.26–0.48)	<0.0001
Protozoa	0.37 (0.26–0.51)	<0.0001	0.51 (0.36–0.73)	<0.0001
Helminth	0.26 (0.19–0.35)	<0.0001	0.37 (0.27–0.52)	<0.0001
Poly–parasitism – any	0.31 (0.19–0.51)	<0.0001	0.43 (0.26–0.71)	0.001
Protozoa plus helminth	0.29 (0.17–0.50)	<0.0001	0.36 (0.20–0.65)	0.001
Helminth plus helminth	0.33 (0.14–0.79)	0.013	0.52 (0.21–1.25)	0.143
Soil–transmitted helminths only	0.39 (0.23–0.68)	0.001	0.43 (0.24–0.77)	0.004

OR=odds ratio. *Adjusted for gender, age, residence, education level, body mass index; **Adjusted for gender, age, residence, education level, body mass index, and comorbidities.

## 4 Discussion

This study demonstrates for the first time that co-infection with enteric parasites, both protozoa and helminths, is associated with lower odds of developing severe COVID-19 in African patients. Notably, this association was maintained even after adjusting for sex, age, residency, education level, and presence of comorbid conditions, factors that are commonly associated with COVID-19 severity [2], [3], [4], [5]. Previous reports have demonstrated that helminthic correlates with a lower risk for development of diabetes, and metabolic syndrome in humans [19], [20], [21], [22], [23], [24]. This is consistent with our finding that patients co-infected with parasites had lower proportions of NCDs. This finding suggests that the inverse correlation between parasitic infection and COVID-19 severity in our cohort can be attributed at least partly to decreased NCDs risk. In this study, we noted that NCDs, in particular hypertension, chronic liver disease, overweight, and obesity were all associated with increased risk of COVID-19 severity. Nonetheless, pregnancy, and NCDs such as chronic obstructive lung disease, chronic kidney disease, surgery, and HIV-1 infection appeared not associated with severe COVID-19, most likely due to the smaller sample size of patients with these conditions.

The pathogenesis of severe COVID-19 has been linked to the phenomenon of immune hyperactivation [25], which resembles that of chronic inflammatory condition, such as hypertension, obesity, diabetes, and inflammatory bowel diseases [26], [27], [28]. Lifestyle factors, such as high calorie diet, physical inactivity, and higher standard of living, coupled with decreased rates of helminthic infections, in HICs has been linked with the advent of chronic inflammatory conditions – compatible with the theory of hygiene hypothesis [29,30]. Indeed, several authorities proposed that lack of co-infection with parasites may lead to increased risk of COVID-19 severity in HICs [[11], [12], [13], 29,30]. It is possible, therefore, that parasites mute COVID-19 severity through their effects in modulating systemic immune response. Chronic intestinal parasitic infections are often associated with the development of T helper-2 (TH2)-skewed, alternatively activated macrophages (M2), and type 2 innate lymphoid cells. These responses are accompanied with the induction of cytokines such as IL-4, IL-5, IL-13, and enhanced eosinophilia, and IgE responses [8,9]. Parasite-driven TH-2 responses are important in controlling parasitic infections, as well as play an important role in the repair of tissue damage as a result of parasite infections [8,9]. TH2 immune responses during parasitic infections is also accompanied by the induction of a strong T cell regulatory (Treg) responses that is relevant for survival and chronic persistence of the parasite itself, that may affect responses to heterologous infection [8,9,29]. On the contrary, severe COVID-19 is associated with increased hyperinflammation characterized by increased production of pro-inflammatory cytokines [25]. Thus, persistent parasite-driven TH2, and Treg responses, in turn, may counterbalance overactive TH1 responses, which have been described in severe COVID-19 [13]. In addition, parasite-driven gut microbiome changes may modulate the host's immune response [13]. Thus, it is possible that parasitic infections may affect pathogenesis both through direct modulation of the immune system as well as through an indirect parasite-driven microbiome balance [8,9,13]. Indeed, it has been demonstrated in animal models that enteric helminths can protect against pulmonary viral infections through interaction with microbiota [31]. Paradoxically, lack of hygienic practice in parasite endemic areas of LMICs may increase the risk of transmission and infection of SARS-CoV-2 [29].

The strengths of the current study include its prospective nature. However, our study has some limitations. First, it was not possible to collect stool samples for every consecutive patient which may have resulted in a potential selection bias. Second, stool examination was determined by microscopy only. Although PCR has been shown to be superior to microscopy with increased sensitivity and specificity [32], the presence of very low intensity of infection determined by PCR, might indeed preclude the effects on immune modulation. Third, some of the parasite species-specific associations with COVID-19 severity could not be ascertained in the current study because of small sample sizes/prevalence. Fourth, it is difficult to ascertain whether the cases included in our study can be considered representative of the wider population, given the fact that there is lack of data from the country. Finally, the inclusion of a smaller proportion of critical cases (which are rare in our settings) [33], as compared to other categories within the clinical spectrum of COVID-19 in our cohort may potentially bias the results. Confounding through other treatments the patients may have received on their COVID-19 severity in our cohorts is negligible, as very few patients (*n* = 5) took remdesivir. In addition, none of the patients co-infected with parasites included in the cohort did receive anti-parasitic drug ivermectin, that has been shown to exhibit in-vitro activity against SARS-CoV-2 [34].

In conclusion, our study is the first to show a significant inverse correlation between the presence of intestinal parasites and COVID-19 severity, suggesting that parasite co-infection, with both protozoa and helminths, may protect against progression to severe COVID-19. This is corroborated by the observed low COVID-19 fatality rate in LMIC settings where parasitic infections are endemic [11,12]. Nonetheless, causality cannot be inferred from the current study design. Thus, more evidence corroborating this association is needed, with corroboration in other LMIC settings, and studies with larger sample sizes that permit testing of possible interplay between the parasite microbiome on COVID-19 severity [13]. Unraveling the parasite-modulated mechanisms underlying severe COVID-19 offers avenues for novel preventive and therapeutic interventions. Moreover, parasitic infections might have repercussions for efficacy of current COVID-19 vaccines administered in Africa through COVAX initiative [35], and could lead to alternative approaches, such as: first deworm, than vaccinate. Studies in this area are recommended.

## 5 Contributors

DW, VH, and TRW were involved in funding acquisition, and conceived and designed the study. TG, ZGA, YK, AG, GT, MA, KLE, TGH, and FKM followed the patients, collected clinical samples and data. TG, AH, GT, and SA did the laboratory analysis. HEA and AD collected data. GT, MT, and EA were involved in funding acquisition, and study design. BCU and HHDFS were involved in funding acquisition, study design, data interpretation, and draft writing and review. DW, and TRW wrote the first draft of the manuscript; both were responsible for access and data accession of the raw data. All authors contributed to data interpretation, critically reviewed the manuscript, and approved the final version for submission.

## Declaration of Competing Interest

DW is European and Developing Countries Clinical Trials Partnership (EDCTP) Senior Research Fellow, and received funding for EvaLAMP project on Leishmaniasis Diagnostics; he serves as Strategic and Scientific Advisory Board of the Research Networks for Health Innovations in Sub-Saharan Africa (German Federal Ministry of Education and Research), and has received an honorarium for lectures and presentations from the Ethiopian Ministry of Science and Higher Education. VH received grants from Netherlands organization for Health Research and Development, VaillantFonds, and she serves as Gilead advisory board, and has received an honorarium from Medtalks, and Gilead. In addition, she serves as head of expertise group for Federal Medical Specialists, and is reviewer for COVID-19 grants for Netherlands organization for Health Research and Development. TRW is employee of PharmAccess Foundation, is Board Member of Mondial Diagnostics, and Advisory Board member of Healthinc, The Netherlands. All other authors have no declarations to disclose.

## Appendix Supplementary materials

Image, application 1

Image, application 2
