# Supplementary material for: Effect of co-infection with intestinal parasites on COVID-19 severity: A prospective observational cohort study
Source: eClinicalMedicine. 2021 Jul 31;39:101054. doi: 10.1016/j.eclinm.2021.101054 (PMC8324426; doi:10.1016/j.eclinm.2021.101054)
Supplement: Supplementary file 1 [file mmc1.docx]

**APPENDIX: SUPPLEMENTARY MATERIAL**

**Page 1:** Supplemental Table 1 – Clinical features among COVID–19 patients by severity category

**Page 3:** Supplemental Table 2 – Clinical features of COVID–19 patients without or with non-communicable disease (NCD)

**Page 5:** *Supplemental Figure 1:* Proportion of COVID-19 cases with parasite co-infection (poly-parasites, or combined protozoa-helminth, or helminth-helminth, and specicies-specific) with the different stages of COVID-19

**Page 6.** Supplemental Table 3 – Factors associated with non-communicable diseases (≥ 1) among COVID-19 patients

**Page 7:** Supplemental Table 4 – The STROBE Statement: Checklist of items that should be addressed in reports of observational studies
