# Supplementary material for: Effect of co-infection with intestinal parasites on COVID-19 severity: A prospective observational cohort study
Source: eClinicalMedicine. 2021 Jul 31;39:101054. doi: 10.1016/j.eclinm.2021.101054 (PMC8324426; doi:10.1016/j.eclinm.2021.101054)
Supplement: Supplementary file 2 [file mmc2.docx]

**Supplemental Table 1 – Clinical features among COVID–19 patients by severity category**

| **Characteristics** | **COVID-19 severity category** | | | | **p-Value** |
| --- | --- | --- | --- | --- | --- |
|  | **Asymptomatic**  **(n=272)** | **Mild/moderate**  **symptomatic**  **(n=224)** | **Severe**  **(n=218)** | **Critical**  **(n=37)** |  |
| **Socio–demographic features:** |  |  |  |  |  |
| Gender |  |  |  |  |  |
| Male | 166 (61.0) | 132 (58.9) | 159 (72.9) | 23 (62.2) | 0.004 |
| Female (not pregnant) | 104 (38.2) | 87 (38.8) | 52 (23.9) | 14 (37.8) |  |
| Female (pregnant) | 2 (0.7) | 5 (2.2) | 7 (3.2) | 0 (0.0) |  |
| Age in years | 30 (24–38) | 32 (26–45) | 52 (40–65) | 50 (37–64) | 0.0001 |
| Age group [years] |  |  |  |  |  |
| < 24 | 63 (23.1) | 27 (12.1) | 1 (0.5) | 1 (2.7) | <0.0001 |
| 24 – 44 | 169 (62.1) | 140 (62.5) | 76 (34.9) | 13 (35.1) |  |
| 45 – 59 | 33 (12.1) | 39 (17.4) | 57 (26.2) | 11 (29.7) |  |
| ≥ 60 | 7 (2.6) | 18 (8.0) | 84 (38.5) | 12 (32.4) |  |
| Residence |  |  |  |  |  |
| Rural | 79(29.0) | 44 (19.6) | 15 (6.9) | 3 (8.1) | <0.0001 |
| Urban | 183 (67.3) | 176 (78.6) | 201 (92.2) | 33 (89.2) |  |
| Undetermined | 10 (3.7) | 4 (1.8) | 2 (0.9) | 1 (2.7) |  |
| Education level |  |  |  |  |  |
| ≤ Primary | 83 (30.5) | 41 (18.3) | 71 (32.6) | 20 (54.1) | <0.0001 |
| Secondary | 91 (33.5) | 54 (24.1) | 53 (24.3) | 5 (13.5) |  |
| College/university | 98 (36.0) | 129 (57.6) | 94 (43.1) | 12 (32.4) |  |
| Occupation |  |  |  |  |  |
| Unemployed | 99 (36.4) | 63 (28.1) | 72 (33.0) | 16 (43.2) | 0.001 |
| Employed | 157 (57.7) | 120 (53.6) | 125 (57.3) | 19 (51.4) |  |
| Health care worker | 16 (5.9) | 41 (18.3) | 21 (9.6) | 2 (5.4) |  |
| **Clinical symptoms and signs:** |  |  |  |  |  |
| Fever | 0 (0.0) | 81 (36.2) | 138 (63.3) | 29 (78.4) | <0.0001 |
| Dyspnea | 0 (0.0) | 26 (11.7) | 145 (66.5) | 28 (75.7) | <0.0001 |
| Cough (any type) | 0 (0.0) | 122 (54.5) | 183 (83.9) | 34 (91.9) | <0.0001 |
| Hemoptysis | 0 (0.0) | 7 (3.1) | 11 (5.1) | 3 (8.1) | <0.0001 |
| Chest pain | 0 (0.0) | 8 (3.6) | 30 (13.8) | 9 (24.3) | <0.0001 |
| Sore throat | 0 (0.0) | 25 (11.2) | 39 (17.9) | 6 (16.2) | <0.0001 |
| Head ache | 1 (0.4) | 89 (39.7) | 114 (52.3) | 19 (51.4) | <0.0001 |
| Nasal congestion | 0 (0.0) | 26 (11.6) | 15 (6.9) | 1 (2.7) | <0.0001 |
| Loss of smell and/or taste | 0 (0.0) | 46 (20.5) | 10 (4.5) | 3 (8.1) | <0.0001 |
| Nausea/vomiting | 0 (0.0) | 21 (9.4) | 44 (20.2) | 11 (29.7) | <0.0001 |
| Abdominal pain | 2 (0.7) | 11 (4.9) | 25 (11.5) | 6 (16.2) | <0.0001 |
| Diarrhea | 0 (0.0) | 8 (3.6) | 27 (12.4) | 4 (10.8) | <0.0001 |
| Myalgia | 0 (0.0) | 27 (12.1) | 70 (32.1) | 15 (40.5) | <0.0001 |
| Body mass index |  |  |  |  |  |
| 18.5–24.9 | 260 (95.6) | 203 (90.6) | 137 (62.8) | 22 (59.5) | <0.0001 |
| <18.5 | 1 (0.4) | 1 (0.5) | 0 (0.0) | 0 (0.0) |  |
| 25.0–29.9 | 11 (4.0) | 16 (7.1) | 43 (19.7) | 6 (16.2) |  |
| >30.0 | 0 (0.0) | 4 (1.8) | 38 (17.4) | 9 (24.3) |  |
| Temperature > 37.3 ^0^C | 1 (0.4) | 6 (2.7) | 30 (13.8) | 9 (24.3) | <0.0001 |
| Temperature | 36.0 (36.0–36.6) | 36.0 (36.0–36.7) | 36.7 (36.0–37.0) | 37.0 (36.0–37.1) | 0.0001 |
| Systolic blood pressure (mmHg) | 115 (109–125) | 115 (110–124) | 123 (110–137) | 125 (106–137) | 0.0001 |
| Diastolic blood pressure (mmHg) | 75 (67–80) | 76 (70–80) | 72 (68–80) | 70 (66–80) | 0.0607 |
| Respiratory rate (breaths/minute) | 21 (19–23) | 22 (20–23) | 28 (24–30) | 32 (26–38) | 0.0001 |
| Heart rate (beats/minute) | 80 (75–88) | 85 (76–90) | 90 (81–105) | 92 (86–102) | 0.0001 |
| **Laboratory data*** |  |  |  |  |  |
| Lymphocyte, x10^9^/L | 1.6 (1.4–2.1) | 1.2 (0.8–1.6) | 1.2 (0.7–1.5) | 0.9 (0.3–1.8) | 0.0287 |
| Haematocrite, % | 41.6 (35.4–45.9) | 42.7 (34.2–44.5) | 44.5 (41.1–47.0) | 44.6 (41.1–48.7) | 0.0134 |
| Platelet count, x10^9^ /L | 238 (176–310) | 207 (158–288) | 213 (154–288) | 223 (173–303) | 0.537 |
| Alanine aminotransferase concentration, U/L | 30 (14–49) | 32 (17–55) | 40 (27–70) | 41 (25–84) | 0.2047 |
| Creatinine concentration, mg/dL | 0.56 (0.43–0.77) | 0.69 (0.60–0.89) | 0.90 (0.70–1.08) | 0.82 (0.70–0.92) | 0.0001 |
| **Comorbidities** |  |  |  |  |  |
| Comorbidity (at least 1) | 29 (10.7) | 33 (14.7) | 132 (60.6) | 23 (62.2) | <0.0001 |
| Non–communicable disease (NCDs) comorbidities | 14 (5.2) | 27 (12.1) | 117 (53.6) | 21 (56.8) | <0.0001 |
| Diabetes | 8 (2.94) | 16 (7.1) | 61 (28.0) | 12 (32.4) | <0.0001 |
| Hypertension | 3 (1.1) | 10 (4.5) | 62 (28.4) | 12 (32.4) | <0.0001 |
| Cardio–vascular diseases | 1 (0.4) | 3 (1.3) | 14 (6.4) | 2 (5.4) | <0.0001 |
| Chronic obstructive lung diseases, incl. asthma | 5 (1.8) | 7 (3.1) | 9 (4.1) | 2 (5.4) | 0.301 |
| Chronic liver disease | 1 (0.4) | 2 (0.9) | 5 (2.3) | 1 (2.7) | 0.144 |
| Chronic kidney disease | 3 (1.1) | 1 (0.5) | 7 (3.2) | 0 (0.0) | 0.113 |
| Surgical cases | 7 (2.6) | 4 (1.8) | 4 (1.8) | 0 (0.0) | 0.929 |
| Communicable disease comorbidities |  |  |  |  |  |
| HIV | 5 (1.8) | 2 (0.9) | 9 (4.1) | 1 (2.7) | 0.122 |
| Tuberculosis | 0 (0.0) | 0 (0.0) | 0 (0.0) | 1 (2.7) | 0.049 |
| **Outcomes** |  |  |  |  |  |
| Admission to ICU | 0 (0.0) | 1 (0.5) | 18 (8.3) | 37 (100.0) | <0.0001 |
| Supplemental oxygen | 0 (0.0) | 3 (1.3) | 203 (93.1) | 37 (100.0) | <0.0001 |
| Invasive mechanical ventilation | 0 (0.0) | 0 (0.0) | 16 (7.3) | 37 (100.0) | <0.0001 |
| Death | 1 (0.4) | 1 (0.5) | 4 (1.8) | 5 (13.5) | <0.0001 |

Data are expressed as n (%) or median (IQR). p values are from χ² test, or Fisher’s Exact test (for categorical variables), and Mann-Whitney U, or Kruskal-Wallis tests (for continuous variables), as appropriate.

**Supplemental Table 2 – Clinical features of COVID–19 patients without or with non-communicable disease (NCD)**

| **Characteristics** | **No NCD**  **n=572**  **(76.2%)** | **With NCD (≥ 1)**  **n=179**  **(23.8%)** | **p-Value** |
| --- | --- | --- | --- |
| **Socio–demographic features:** |  |  |  |
| Gender |  |  |  |
| Male | 355 (62.1) | 125 (69.8) | 0.033 |
| Female (not pregnant) | 203 (35.5) | 54 (30.2) |  |
| Female (pregnant) | 14 (2.5) | 0 (0.0) |  |
|  |  |  |  |
| Age in years | 33 (26–42) | 57 (45–67) | <0.00001 |
| Age group [years] |  |  |  |
| < 24 | 85 (14.9) | 7 (3.9) | 0.0001 |
| 24 – 44 | 360 (63.1) | 37 (20.7) |  |
| 45 – 59 | 86 (15.1) | 54 (30.2) |  |
| ≥ 60 | 40 (7.0) | 81 (45.3) |  |
| Residence |  |  |  |
| Rural | 123(21.5) | 18 (10.1) | 0.0005 |
| Urban | 435 (76.1) | 158 (88.3) |  |
| Undetermined | 14 (2.5) | 3 (1.7) |  |
| Education level |  |  |  |
| ≤ Primary | 137 (24.0) | 78 (43.6) | <0.0001 |
| Secondary | 162 (28.3) | 41 (22.9) |  |
| College/university | 273 (47.7) | 60 (33.5) |  |
| Occupation |  |  |  |
| Unemployed | 173 (30.2) | 77 (43.0) | 0.001 |
| Employed | 329 (57.5) | 92 (51.4) |  |
| Health care worker | 70 (12.2) | 10 (5.6) |  |
| **Clinical symptoms and signs:** |  |  |  |
| Fever | 148 (25.9) | 100 (55.9) | <0.0001 |
| Dyspnea | 92 (16.1) | 107 (59.8) | <0.0001 |
| Cough (any type) | 201 (35.1) | 138 (77.1) | <0.0001 |
| Non–productive cough | 84 (14.7) | 64 (35.8) | 0.0001 |
| Productive cough | 117 (20.5) | 74 (41.3) |  |
| Hemoptysis | 13 (2.3) | 8 (4.5) | 0.120 |
| Chest pain | 18 (3.2) | 29 (16.2) | <0.0001 |
| Sore throat | 44 (7.7) | 26 (14.5) | 0.006 |
| Head ache | 146 (25.5) | 77 (43.0) | <0.0001 |
| Nasal congestion | 31 (5.4) | 11 (6.2) | 0.712 |
| Loss of smell and/or taste | 53 (9.3) | 6 (3.4) | 0.010 |
| Nausea/vomiting | 46 (8.0) | 30 (16.8) | 0.001 |
| Abdominal pain | 23 (4.0) | 21 (11.7) | <0.0001 |
| Diarrhea | 21 (3.7) | 18 (10.1) | 0.001 |
| Myalgia | 58 (10.1) | 54 (30.2) | <0.0001 |
| Body mass index |  |  |  |
| 18.5–24.9 | 561 (98.1) | 61 (34.1) | <0.0001 |
| <18.5 | 1 (0.2) | 1 (0.6) |  |
| 25.0-29.9 | 9 (1.6) | 67 (37.4) |  |
| ≥30.0 | 1 (0.2) | 50 (27.9) |  |
| Temperature > 37.3 ^0^C | 19 (3.3) | 27 (15.1) | <0.0001 |
| Temperature | 36.0 (36.0–36.7) | 36.7 (36.0–37.0) | <0.00001 |
| Systolic blood pressure (mmHg) | 115 (109–124) | 130 (118–142) | <0.00001 |
| Diastolic blood pressure (mmHg) | 75 (68–80) | 77 (70–85) | 0.0002 |
| Respiratory rate (breaths/minute) | 22 (20–24) | 26 (22–31) | <0.00001 |
| Heart rate (beats/minute) | 85 (76–90) | 90 (80–103) | <0.00001 |
| **Laboratory data*** |  |  |  |
| Lymphocyte, x10^9^/L | 1.2 (0.7–1.7) | 1.2 (0.7–1.5) | 0.4231 |
| Haematocrite, % | 43.1 (38.8–46.6) | 44.5 (41.9–47.0) | 0.0684 |
| Platelet count, x10^9^ /L | 213 (152–279) | 217 (170–297) | 0.2921 |
| Alanine aminotransferase concentration, U/L | 40 (19–65) | 38 (27–66) | 0.4627 |
| Creatinine concentration, mg/dL | 0.72 (0.60–0.95) | 0.90 (0.73–1.08) | 0.0001 |
| **Outcomes** |  |  |  |
| Severe COVID-91 clinical status | 117 (20.5) | 138 (77.1) | <0.0001 |
| Admission to ICU | 22 (3.9) | 34 (19.0) | <0.0001 |
| Supplemental oxygen | 115 (20.1) | 128 (71.5) | <0.0001 |
| Invasive mechanical ventilation | 18 (3.2) | 35 (19.5) | <0.0001 |
| Death | 6 (1.1) | 5 (2.8) | 0.090 |
| Data are expressed as n (%) or median (IQR). p values are from χ² test, or Fisher’s Exact (for categorical variables), and Mann-Whitney U, or Kruskal-Wallis tests (for continuous variables), as appropriate. | | | |

***Supplemental Figure 1:* Proportion of COVID-19 cases with parasite co-infection (poly-parasites, or combined protozoa-helminth, or helminth-helminth, and specicies-specific) with the different stages of COVID-19.** Error bars indicate 95% CI. p values (estimated by Kruskal-Wallis rank test) presented in the table below figure.


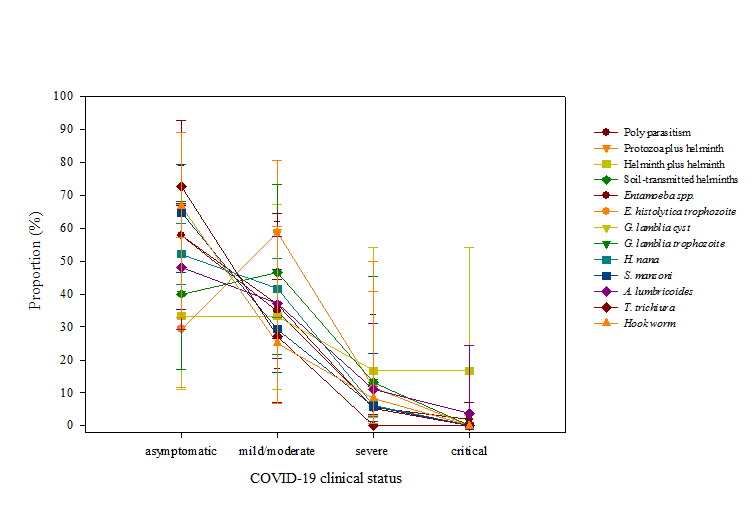


| **Parasite** | **p-Value** |
| --- | --- |
| Poly-parasitism | 0.0001 |
| Protozoa plus helminth | 0.0001 |
| Helminth plus helminth | 0.0551 |
| Soil transmitted helminths | 0.0037 |
| *Entamoeba spp.* cyst | 0.0001 |
| *E. histolytica trophozoite* | 0.0513 |
| *G. lamblia cyst* | 0.2482 |
| *G. lamblia trophozoite* | 0.3126 |
| *H. nana* | 0.0001 |
| *S. mansoni* | 0.0010 |
| *A. lumbricoides* | 0.1899 |
| *T. trichiura* | 0.0479 |
| Hook worm | 0.1322 |
| *Taenia spp.* | 0.1512 |

**Supplemental Table 3 – Factors associated with non-communicable diseases (≥ 1) among COVID-19 patients**

|  | **Characteristic** | **Univariable model** | |  | | **Multivariable model** | |  |
| --- | --- | --- | --- | --- | --- | --- | --- | --- |
|  |  | **Unadjusted OR (95% CI)** | **p value** |  | | **Adjusted OR**  **(95% CI)** | **p-Value** |  |
|  | Gender |  |  |  |  | |  |  |
|  | Male | 1 | .. |  | 1 | | .. |  |
|  | Female (non-pregnant) | 0.76 (0.53–1.09) | 0.130 |  | .. | | .. |  |
|  | Female (pregnant) | 1.0 | .. |  | .. | | .. |  |
|  | Age (≥60 years vs. <60) | 10.99 (7.11–16.70) | <0.0001 |  | **7.95 (4.56–13.87)** | | **<0.0001** |  |
|  | Rural residence | 0.50 (0.32–0.77) | 0.002 |  | | 0.82 (0.49–1.39) | 0.465 |  |
|  | Education level |  |  |  | |  |  |  |
|  | ≤ Primary | 1 | .. |  | | 1 | .. |  |
|  | Secondary | 0.44 (0.29–0.69) | <0.0001 |  | | 0.92 (0.51–1.67) | 0.782 |  |
|  | College/university | 0.39 (0.26–057) | <0.0001 |  | | 0.58 (0.32–1.05) | 0.072 |  |
|  | Occupation |  |  |  | |  |  |  |
|  | Unemployed | 1 | .. |  | | 1 | .. |  |
|  | Employed | 0.63 (0.44–0.90) | 0.010 |  | | 0.84 (0.52–1.37) | 0.494 |  |
|  | Health care workers | 0.32 (0.16–0.66) | 0.002 |  | | 0.38 (0.13–1.11) | 0.077 |  |
|  | Body mass index |  |  |  | |  |  |  |
|  | 18.5–24.9 | 1 | .. |  | | 1 | .. |  |
|  | <18.5 | 1.71 (0.18–16.59) | 0.644 |  | | 2.40 (0.24–24.30) | 0.459 |  |
|  | 25.0–29.9 | 23.59 (11.55–48.18) | <0.0001 |  | | 29.76 (12.9–68.47) | <0.0001 |  |
|  | ≥30.0 | 1.00 | .. |  | | 1.00 | .. |  |
|  | Parasite co–infection** |  |  |  | |  |  |  |
|  | Any parasite (at least 1) | 0.24 (0.15–0.37) | <0.0001 |  | | **0.35 (0.21–0.60)** | **<0.0001** |  |
|  | Protozoa | 0.45 (0.27–0.73) | 0.001 |  | | 0.72 (0.39–1.36) | 0.320 |  |
|  | Helminth | 0.15 (0.08–0.29) | <0.0001 |  | | **0.21 (0.10–0.43)** | **<0.0001** |  |
|  | Poly–parasitism – any | 0.19 (0.07–0.54) | 0.002 |  | | **0.25 (0.08–0.85)** | **0.026** |  |
|  | Protozoa plus helminth | 0.19 (0.06–0.62) | 0.006 |  | | **0.20 (0.05–0.82)** | **0.026** |  |
|  | Helminth plus helminth | 1.00 (..–..) | .. |  | | 1.00 | .. |  |
|  | Soil–transmitted helminths only | 0.44 (0.18–1.05) | 0.063 |  | | .. | .. |  |
|  | Species-specific |  |  |  | |  |  |  |
|  | *Entamoeba cyst spp.* | 0.28 (0.14–0.55) | <0.0001 |  | | **0.41 (0.19–0.92)** | **0.031** |  |
|  | *Entamoeba histolytica trophozoite* | 1.34 (0.47–3.86) | 0.586 |  | | .. | .. |  |
|  | *Giardia lamblia cyst* | 1.07 (0.29–3.98) | 0.924 |  | | .. | .. |  |
|  | *Giardia lamblia trophozoite* | 0.80 (0.22–2.85) | 0.725 |  | | .. | .. |  |
|  | *Hymenolopis nana* | 0.07 (0.02–0.23) | <0.0001 |  | | **0.09 (0.02–0.34)** | **<0.0001** |  |
|  | *Schistosoma mansoni* | 0.09 (0.01–0.68) | 0.019 |  | | **0.12 (0.01–0.99)** | **0.049** |  |
|  | *Ascaris lumbricoides* | 0.39 (0.12–1.31) | 0.127 |  | | .. | .. |  |
|  | *Trichuris trichura* | 0.32 (0.04–2.48) | 0.273 |  | | .. | .. |  |
|  | Hook worm | 1.07 (0.29–3.98) | 0.924 |  | | .. | .. |  |
|  | *Taenia spp.* | 1.00 (..–..) | .. |  | | .. | .. |  |
|  | OR=odds ratio. *Adjusted for age, residence, education level, occupation, and body mass index. | | | | | | |  |

**Supplemental Table 4 – The STROBE Statement: Checklist of items that should be addressed in reports of observational studies**

|  | **Item** | **Recommendation** | **Reported on manuscript page** |
| --- | --- | --- | --- |
| **Title and abstract** |  |  |  |
|  | 1 | (a) Indicate the study’s design with a commonly used term in the title or the abstract  (b) Provide in the abstract an informative and balanced summary of what was done and what was found | 1  2 |
| **Introduction** |  |  |  |
| Background/ rationale | 2 | Explain the scientific background and rationale for the investigation being reported | 4 |
| Objectives | 3 | State specific objectives, including any pre-specified hypotheses | 4, 5 |
| **Methods** |  |  |  |
| Study design | 4 | Present key elements of study design early in the paper | 5 |
| Setting | 5 | Describe the setting, locations, and relevant dates, including periods of recruitment, exposure, follow-up, and data collection | 4, 5 |
| Participants | 6 | (a)..Cohort study—Give the eligibility criteria, and the sources and methods of selection of participants. Describe methods of follow-up  Case-control study—Give the eligibility criteria, and the sources and methods of case ascertainment and control selection. Give the rationale for the choice of cases and controls Cross-sectional study—Give the eligibility criteria, and the sources and methods of selection of participants  (b) Cohort study—For matched studies, give matching criteria and number of exposed and unexposed  Case-control study—For matched studies, give matching criteria and the number of controls per case | 4, 5 |
| Variables | 7 | Clearly define all outcomes, exposures, predictors, potential confounders, and effect modifiers. Give diagnostic criteria, if applicable | 5 |
| Data sources/ measurement | 8 | For each variable of interest, give sources of data and details of methods of assessment (measurement).  Describe comparability of assessment methods if there is more than one group | 5 |
| Bias | 9 | Describe any efforts to address potential sources of bias | 5 |
| Study size | 10 | Explain how the study size was arrived at | 5 |
| Quantitative variables | 11 | Explain how quantitative variables were handled in the analyses. If applicable, describe which groupings were chosen, and why | 5 |
| Statistical methods | 12 | (a) Describe all statistical methods, including those used to control for confounding  (b) Describe any methods used to examine subgroups and interactions  (c) Explain how missing data were addressed  (d) Cohort study—If applicable, explain how loss to follow-up was addressed  Case-control study—If applicable, explain how matching of cases and controls was  addressed  Cross-sectional study—If applicable, describe analytical methods taking account of  sampling strategy  (e) Describe any sensitivity analyses | 5 |
| **Results** |  |  |  |
| Participants | 13 | (a) Report the numbers of individuals at each stage of the study—e.g., numbers potentially eligible, examined for eligibility, confirmed eligible, included in the study, completing follow-up, and analysed  (b) Give reasons for non-participation at each stage  (c) Consider use of a flow diagram | 5  Appendix p1 |
| Descriptive data | 14 | (a) Give characteristics of study participants (e.g., demographic, clinical, social) and information on exposures and potential confounders  (b) Indicate the number of participants with missing data for each variable of interest  (c) Cohort study—Summarise follow-up time (e.g., average and total amount) | 5, 6 |
| Outcome data | 15 | Cohort study—Report numbers of outcome events or summary measures over time  Case-control study—Report numbers in each exposure category, or summary measures of exposure  Cross-sectional study—Report numbers of outcome events or summary measures | 5, 6 |
| Main results | 16 | (a)..Give unadjusted estimates and, if applicable, confounder adjusted estimates and their precision (e.g., 95% confidence interval).  Make clear which confounders were adjusted for and why they were included  (b)..Report category boundaries when continuous variables were categorized  (c) If relevant, consider translating estimates of relative risk into absolute risk for a meaningful time period | 5, 6 |
| Other analyses | 17 | Report other analyses done—e.g., analyses of subgroups and interactions, and sensitivity analyses | 6 |
| **Discussion** |  |  |  |
| Key results | 18 | Summarise key results with reference to study objectives | 6, 7 |
| Limitations | 19 | Discuss limitations of the study, taking into account sources of potential bias or imprecision. Discuss both direction and magnitude of any potential bias | 7 |
| Interpretation | 20 | Give a cautious overall interpretation of results considering objectives, limitations, multiplicity of analyses, results from similar studies, and other relevant evidence | 7 |
| Generalisability | 21 | Discuss the generalisability (external validity) of the study results | 7 |
| **Other information** |  |  |  |
| Funding | 22 | Give the source of funding and the role of the funders for the present study and, if applicable, for the original study on which the present article is based | 8 |
